# Multicentre prospective study to evaluate effectiveness and safety of gel-forming and hyaluronic-acid containing chewable tablets as add-on treatment in patients with gastroesophageal reflux disease (GERD) symptoms and unsatisfying proton pump inhibitor therapy

**DOI:** 10.1186/s12876-023-02946-6

**Published:** 2023-09-06

**Authors:** Manfred Gross, Dennis Neuschwander, Lisa Steffens, Jörn Thomsen, Kristina Röschmann-Doose

**Affiliations:** 1 Department of Internal Medicine, Internistisches Klinikum München Süd, 81379 Gastroenterology, München, Germany; 2 GCP-Service International Ltd. & Co. KG, 28359 Bremen, Germany; 3 G. Pohl-Boskamp GmbH & Co. KG, 25551 Hohenlockstedt, Germany

**Keywords:** Gastroesophageal reflux disease (GERD), Proton pump inhibitor (PPI), Bioadhesive gel-forming tablet, Hyaluronic acid

## Abstract

**Background:**

Gastroesophageal reflux disease (GERD) is a common disease which in the majority of patients is treated with proton pump inhibitors (PPI). However, up to 45% of the patients remain symptomatic on a standard dose of PPI. This study investigated the effectiveness and safety of an add-on therapy with the gel-forming chewable tablet Sobrade® in patients unsatisfied with PPI treatment. The bioadhesive gel covers the oesophagus and thereby protects the mucosa from reflux events.

**Methods:**

47 patients with symptomatic GERD despite PPI treatment participated in this study. The gel-forming tablets were taken up to four times daily after meals and prior to bedtime. Severity and frequency of GERD symptoms were evaluated during two onsite visits prior and following 14 days of treatment and used to calculate the GERD score of the Reflux Disease Questionnaire. Furthermore, patients recorded symptoms as well as onset and duration of symptoms relief daily in their electronic dairies. Effectiveness of treatment was analysed using non-parametric paired Wilcoxon test. In addition, anchor-based minimal important differences (MID) were assessed.

**Results:**

Treatment resulted in significant reduction of GERD symptoms. Severity and frequency of 8 of the 9 assessed symptoms improved significantly during the treatment phase whereby most pronounced improvement was observed for heartburn. In agreement, all three subscales of the GERD score improved significantly. MID results suggest that patients considered a mean improvement of symptoms > 30% of initial severity as beneficial. Self-assessments by patients revealed first significant improvements of symptoms like heartburn and regurgitation from day 5 of treatment onwards. 49% of patients reported relief of symptoms within 15 min which lasted on average for 3.5 h. During the study no treatment emergent adverse events were reported and in 98% of all cases tolerability of the product was rated as very good or good.

**Conclusions:**

This study revealed a pronounced improvement of the symptoms after add-on treatment with the gel-forming medical device. The very good safety and tolerability profile indicate an advantageous risk-benefit ratio.

**Trial registration:**

This non-interventional study was prospectively positively evaluated by the responsible ethic-committees.

**Supplementary Information:**

The online version contains supplementary material available at 10.1186/s12876-023-02946-6.

## Introduction

Gastroesophageal reflux disease (GERD) is a very common disease. Approximately 10–20% of the adult population in the Western world (Western Europe and North America) are affected [1–4]. Clinically, it is diagnosed if the reflux symptoms, mainly heart burn, become a frequent problem (> 2 times a week) with such a severity that the quality of life is impaired. The most common symptoms are heartburn, regurgitation, chest pain, cough or hoarseness, throat pain or burning and sleep disturbances [4].

The pathogenesis of gastroesophageal reflux disease is complex [5]. Exposure of the esophagus to the gastric content causes the symptoms. This exposure arises from an insufficient anti-reflux barrier (increased transient or permanent lower esophageal sphincter relaxations, hiatal hernia) or a reduced ability of the esophagus to clear the refluxate. Changes in epithelial resistance and visceral sensitivity may contribute to the development of symptoms [6, 7]. The most commonly prescribed therapeutics regulate gastric pH (e.g. antacids, H_2_ antagonists, proton pump inhibitors (PPIs) [3, 4, 8, 9]. PPIs are generally very safe drugs but in recent years several potential side effects are discussed [10].

Up to 45% of the patients report a limited therapeutic effect of PPIs with ongoing symptoms despite regular and correct intake of the medication[11]. Partial PPI response, according to the Montreal Consensus, is presence of mild heartburn and/or regurgitation on three or more days/week despite at least four weeks of PPI [12]. The pathophysiology of PPI refractory GERD is heterogeneous including various other conditions such as reflux hypersensitivity of the esophagus, functional heartburn, esophageal motility disorders, gastroparesis, psychiatric comorbidities or insufficient pharmacological effect of the PPI [11, 12].

Nevertheless, medical products can serve as a low risk add-on therapy to the PPI treatment. An add-on therapy with an alginate-based medical device acting as a mechanical reflux barrier was shown to successfully control symptoms associated with GERD [13].

Also, medical devices were developed for treatment of GERD symptoms like heartburn and acid regurgitation which differ in their composition but share a common mode of action [14].

Sobrade^®^ chewable tablet is a low risk medical device and certified as class-I product. Its ingredients xanthan and carbomer are present in similar medical devices and the formation of a bioadhesive barrier to prevent the esophageal mucosa from further GERD induced damages was already confirmed in *in-vitro* and *in-vivo* studies [15, 16]. In addition, hyaluronic acid comprised in the barrier plays a well-known role in the healing-process and its supportive function in re-epithelization of irritated mucosa of GERD patients has been previously reported [17, 18]. This non-interventional clinical study was initiated to show that Sobrade^®^ acts as a symptomatically treatment of GERD symptoms.

The objectives were to evaluate the effectiveness of the medical device for the first time in patients suffering from GERD with unsatisfying PPI treatment for at least one year, when used in accordance with its intended use, the safety/tolerability during the treatment phase and the patients’ and investigators’ satisfaction.

Patients qualified for this study if they suffered from symptoms of reflux disease such as pain or burning feeling behind the breastbone or acid taste in the mouth on at least 2 days per week with at least moderate severity despite PPI treatment for at least one year.

## Material and methods

This multicentre, uncontrolled prospective and open-label clinical study was performed according to § 23b German Medical Device Law and conducted at 2 general practitioners and 2 gastroenterologist sites located in the Federal States of North Rhine-Westphalia and Berlin, Germany. It complied to GCP guidelines, the Declaration of Helsinki and received prospectively, positive evaluation by the independent ethic committees of the medical councils of Nordrhein (File number 2020409) and Westfalen-Lippe (File number 2020-07-f-S) prior to enrolment of the first patient. The clinical phase lasted from January to November 2021 whereby recruitment was affected by the COVID-19 pandemic resulting in an unplanned extension of recruitment by 4 months.

### Patients

Before participating in the study, patients read and signed the informed consent form as well as a declaration on data protection. Patient identities were disguised by using a patient identification number. All patients met the inclusion criteria which were an age > 18 years; diagnosed GERD by the responsible physician with presence of at least 2 of the 3 symptoms “pain behind the breastbone”, “burning feeling behind the breastbone”, “acid taste in the mouth” with at least 3 points for frequency (2–3 days per week) and severity (moderate) on 6-point Likert scale (Appendix A) while receiving PPI treatment for at least one year; ability and willingness to complete self-assessment questionnaires; compliance with contra-indications, precautions for use and warnings mentioned in the Instructions for Use. Females with childbearing potential had to have a negative urine pregnancy test at baseline and had to agree to use a highly effective method of contraception for the duration of the clinical investigation.

Patients were not eligible if one of the following exclusion criteria was met: illness or circumstance that could affect the study purpose in the opinion of the investigator; known or suspected disorders like Barrett’s esophagus or hereditary fructose intolerance; pregnancy or breastfeeding in females; participation in another clinical study within the last 6 months; triple therapy for eradication of *Heliobacter pylori* within 2 weeks prior to the study; employee or direct relative of an employee of the site, CRO or sponsor; prisoner or lawfully kept in an institution.

### Study design and treatment

The study and treatment phase started at Visit 1 and ended at Visit 2 after 14 ± 2 days. At Visit 1, patient’s demographics, body height, weight, concomitant medications and diseases as well as vital signs (body temperature, heart rate and blood pressure) and medical history were documented. Baseline assessments were collected from eligible patients by evaluating the frequency and severity of 9 GERD symptoms over the past seven days “burning feeling behind your breastbone”, “pain behind the breastbone”, “a burning feeling in the center of the upper stomach”, “pain in the center of the upper stomach”, “acid taste in the mouth”, “unpleasant movement of material upwards from the stomach”, “sore throat or hoarseness that is related to heartburn or acid reflux”, “symptoms that caused difficulties in getting a good night’s sleep” and “symptoms prevented eating” on ordinal 6-Point-Likert items (Appendix A). Patients were instructed on the use of an electronic diary (eDiary) for once daily recording of the number of meals and intake of tablets during the study and self-assessment of severity of the following clinical symptoms “heartburn”, “regurgitation”, “hoarseness”, “sleep disturbances” and “symptoms prevented eating” on ordinal 5-point Likert items (Appendix A). Patients received the tablets together with instructions on the correct intake: Sobrade® tablets are chewed slowly until complete dissolution and the forming gel is swallowed. The device should be used after each meal and prior to bedtime up to 4 times daily. Assessment of compliance considered the number of meals consumed daily to calculate the target number of tablets to be taken.

Furthermore, time until onset of symptoms relief as well as duration of symptoms relief were documented whereby for analyses only data from patients with symptoms present on the day of entry were considered (Appendix A).

Visit 2 took place after the 14 ± 2 days treatment period and the investigator evaluated again the frequency and severity of the same 9 GERD symptoms on the ordinal 6-Point-Likert items used for baseline assessments. Furthermore, patient and investigator rated the effectiveness of treatment as well as satisfaction and safety/tolerability of the device on 5-point-Likert items (Appendix A).

### Outcome measures and statistic

Number of patients was based on a sample size calculation assuming a change of 1 from baseline scores for at least one symptom and a standard deviation of 1.7 resulting in 80% power for 48 evaluable patients in case of a paired t-test with 5% two-sided significance level. Furthermore, 50 patients were estimated to be sufficient to have a probability of 90% to detect adverse device effects (ADEs) with 5% incidence under assumption of Poisson distributed data.

Effectiveness was assessed from change of symptom severity and frequency using the numeric versions of the 6-point-Likert items assessed at Visit 1 (baseline) and 2 (final) with higher values of Likert items correspond to more severe (0: not present to 5: severe) and frequent (0: not present to 5: daily) symptoms. Symptoms obtained according to the German Reflux Disease Questionnaire (RDQ) were used to calculate its three subscales which consider mean severity and frequency of acid taste in mouth and movement of material upwards (regurgitation scale), pain or burning behind the breastbone (heartburn scale) or pain or burning in the upper stomach (dyspepsia scale) [19, 20]. The regurgitation and heartburn subscales were summed up in order to determine the GERD Score [19]. In addition, self-assessed symptoms severities were documented daily in patients’ diaries.

Effectiveness was analyzed using non-parametric paired Wilcoxon test for investigator- and self-assessed data without adaptation for multiple testing as data were not normally distributed, however, significance levels do not differ from results of paired t-tests. For diary data, day 1 was used as baseline, although symptoms could have already been alleviated by application of the first tablets. The number of patients with changed symptoms severity from Visit 1 to 2 was analyzed using chi^2^-test. Number of treatment responders was assessed using the following definition: improvement ≥ 1 point of at least one symptom without deterioration of another symptom for “pain behind the breastbone”, “burning behind the breastbone” and “acid taste in mouth”. If not stated otherwise correlation coefficients in the results represent Pearson’s r^2^. Based on the definition by Cohen, r values of 0.1, 0.3 and 0.5 correspond to weak, moderate and strong correlations, respectively [21].

The minimal important difference (MID) was assessed for individual GERD symptoms as well as the RDQ scales regurgitation, heartburn and GERD score against the patient-reported outcomes effectiveness and satisfaction. MID analyses were restricted to significantly correlated parameters (> 0.3 Spearman correlation coefficient) and MID was calculated from change differences obtained for patients grouped based on ratings on 5-point Likert scales, whereby negative and neutral ratings were pooled. MID corresponded to differences of outcomes between patients’ groups with ratings of 1 compared to pooled patients with ratings of -2 to 0. Reliability of the applied anchor-based method was supported by the short treatment phase and similar initial mean values of symptoms in the groups obtained at Visit 1, both features reduce the potential for bias of ratings.

Safety and tolerability of the investigational medicinal product (IMD) with regards to adverse device effects was evaluated in the safety evaluation set (SES) encompassing all patients who received at least one tablet. Effectiveness was analysed using the full analysis set (FAS) which encompassed all eligible patients participated Visit 1 and 2.

Imputed data sets using Baseline observation carried forward (BOCF) including all patients of the SES were used for sensitivity analysis.

All data are presented as mean ± 95% confidence intervals calculated under assumption of t-distributed data.

## Results

### Demographic characteristics

A total of 47 patients at 4 sites participated in the study which was less than planned due to the COVID-19 induced difficulties in recruitment. Forty-two eligible patients completed the study and efficacy data from both visits were available. Complete cases were assigned to the FAS. Mean age of patients was 54.5 years of which 36% were females. PPI treatment lasted on average for 5 years with the majority of patients receiving pantoprazole (66%). Baseline characteristics of participating patients are summarized in Table 1. The majority of patients was recruited at general practitioners, but severity of symptoms did not differ between specialist and general practitioner sites.

**Table 1:** Demographic and clinical characteristics of gastroesophageal reflux disease patients (SES) n (%) or mean (min/max)

Mean age, years (min/max)	54.4 (28/81)
Females	17 (36.2%)
Mean BMI, kg / m^2^ (min/max)	26.7 (21.5/35.2)
Mean Duration of PPI use, years (min/max)	5 (1/21)
Omeprazol	6 (12.8%)
1 x 10 mg	1 (2.1%)
1 x 20 mg	4 (8.5%)
2 x 20 mg	1 (2.1%)
Esomeprazol	9 (19.1%)
1 x 20 mg	4 (8.5%)
1 x 40 mg	2 (4.3%)
2 x 40 mg	2 (4.3%)
1 x 80 mg	1 (2.1%)
Pantoprazol	31 (66.0%)
1 x 20 mg	20 (42.6%)
1 x 40 mg	10 (21.3%)
2 x 40 mg	1 (2.1%)
Missing detailed information on PPI use	1 (2.1%)

### Effectiveness and satisfaction

On Visit 1, severity and frequency of all 9 symptoms were assessed whereby both characteristics were correlated. The symptoms with highest initial severity were “burning behind the breastbone” (3.5 ± 0.2), “pain behind the breastbone” (3.1 ± 0.1) and “acid taste in the mouth” (3.5 ± 0.4). Initially symptom frequencies were highest for “burning behind the breastbone” (3.6 ± 0.2), “pain behind the breastbone” (3.2 ± 0.2) and “acid taste in the mouth” (3.6 ± 0.4). Following 2 weeks treatment period, significant improvement of 8 of the assessed 9 symptoms assessed was observed. Changes of symptom severity and frequency assessed during the visits were comparable with slightly better improvement for severity.

**Fig. 1:**
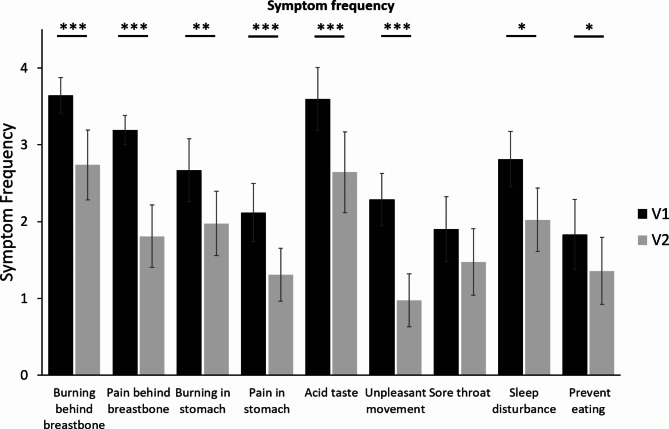
Symptom frequency assessed at Visit 1 and 2 following the two weeks treatment period, mean ± 95% CI; Significant differences are marked by stars, *: p < 0.01, **: p < 0.001, ***: p < 0.0001; N = 42.

Frequencies of “burning behind the breastbone” (3.6 ± 0.2), “pain behind the breastbone” (3.2 ± 0.2) and “acid taste in the mouth” (3.6 ± 0.4) significantly improved at Visit 2 to 2.7 ± 0.5, 1.8 ± 0.4 and 2.6 ± 0.5, respectively (Fig. 1).

**Fig. 2:**
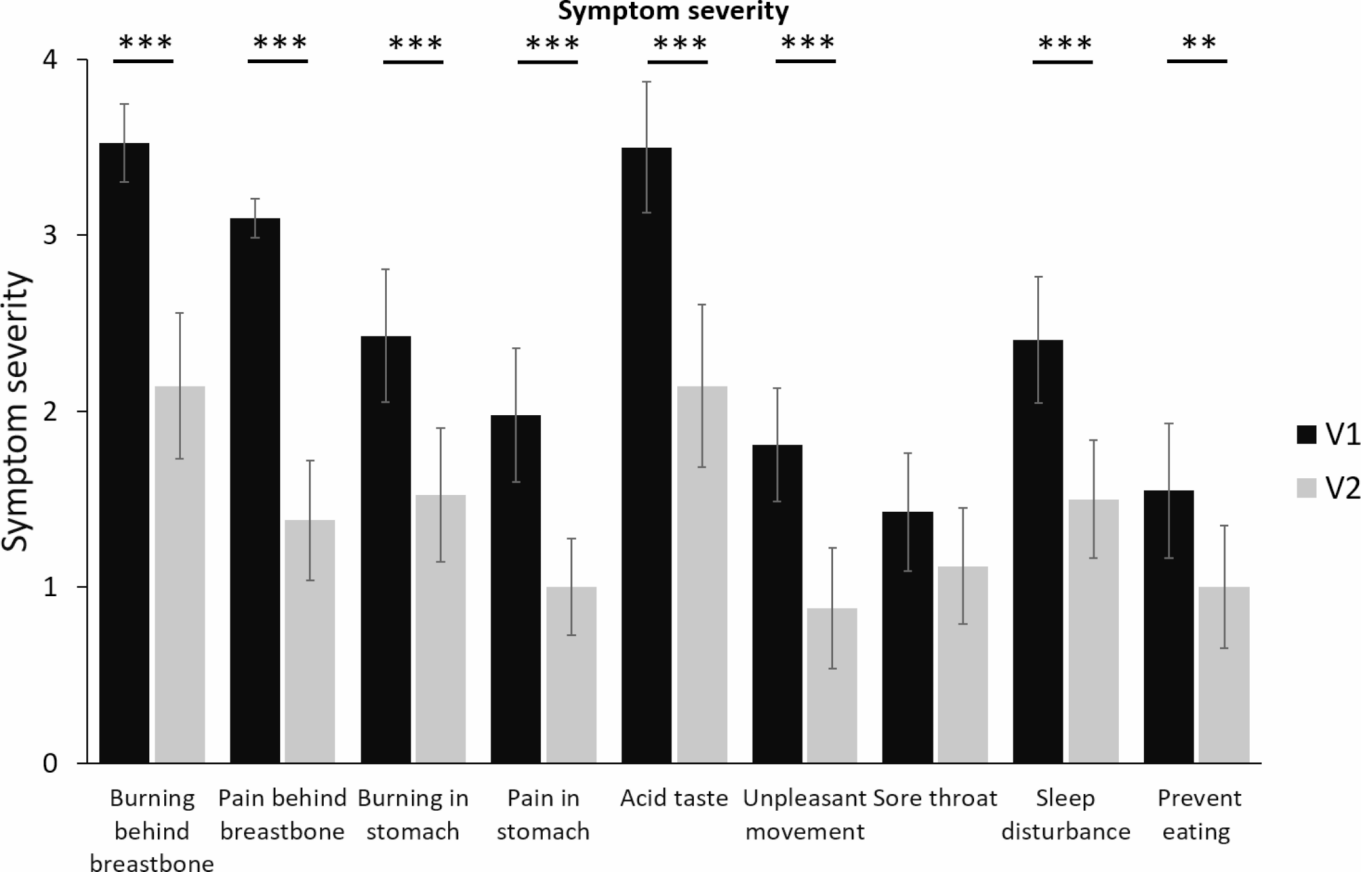
Symptom severity assessed at Visit 1 and 2 following the two weeks treatment period; mean ± 95% CI; Significant differences are marked by stars, *: p < 0.01, **: p < 0.001, ***: p < 0.0001; N = 42.

Most pronounced changes were observed for the severity of the parameters “burning behind the breastbone” and “pain behind the breastbone” which improved by 38.9 and 55.6%, respectively (Fig. 2).

The number of patients with “moderate”, “severe” and “not tolerable” severity for the symptom “burning behind the breastbone” decreased from initially 41 at Visit 1 to 22 at Visit 2. In parallel, number of patients with “mild”, “very mild” and “no symptoms” increased from 1 to 20 (Chi^2^: 22.9, p < 0.0001). Based on the improvement of symptom severity after treatment, 90.5% of patients were considered as responders.

Results were confirmed by the sensitivity analysis using BOCF for patients with missing data from visits which similarly yielded significant differences between Visit 1 and 2.

In line with the single symptoms, scores of RDQ subscales changed during the treatment period. The scores for regurgitation, heartburn and dyspepsia decreased from initial values of 2.8, 3.4 and 2.3, respectively, to 1.7, 2.0 and 1.5 at the final visit (Table 2). In accordance, the GERD score decreased from 6.2 to 3.7 between Visit 1 and 2.

**Table 2:** Subscales of the Reflux Disease Questionnaire and GERD Score at Visit 1 and 2 and results of tests for significance, significant differences between visits are written in bold, mean ± 95% CI.

RDQ	Score		
	Visit 1	Visit 2	p-value
Regurgitation Subscale	2.8 ± 0.3	1.7 ± 0.3	**< 0.0001**
Heartburn Subscale	3.4 ± 0.1	2.0 ± 0.4	**< 0.0001**
Dyspepsia Subscale	2.3 ± 0.3	1.5 ± 0.3	**< 0.0001**
GERD Score	6.2 ± 0.4	3.7 ± 0.7	**< 0.0001**

**Fig. 3:**
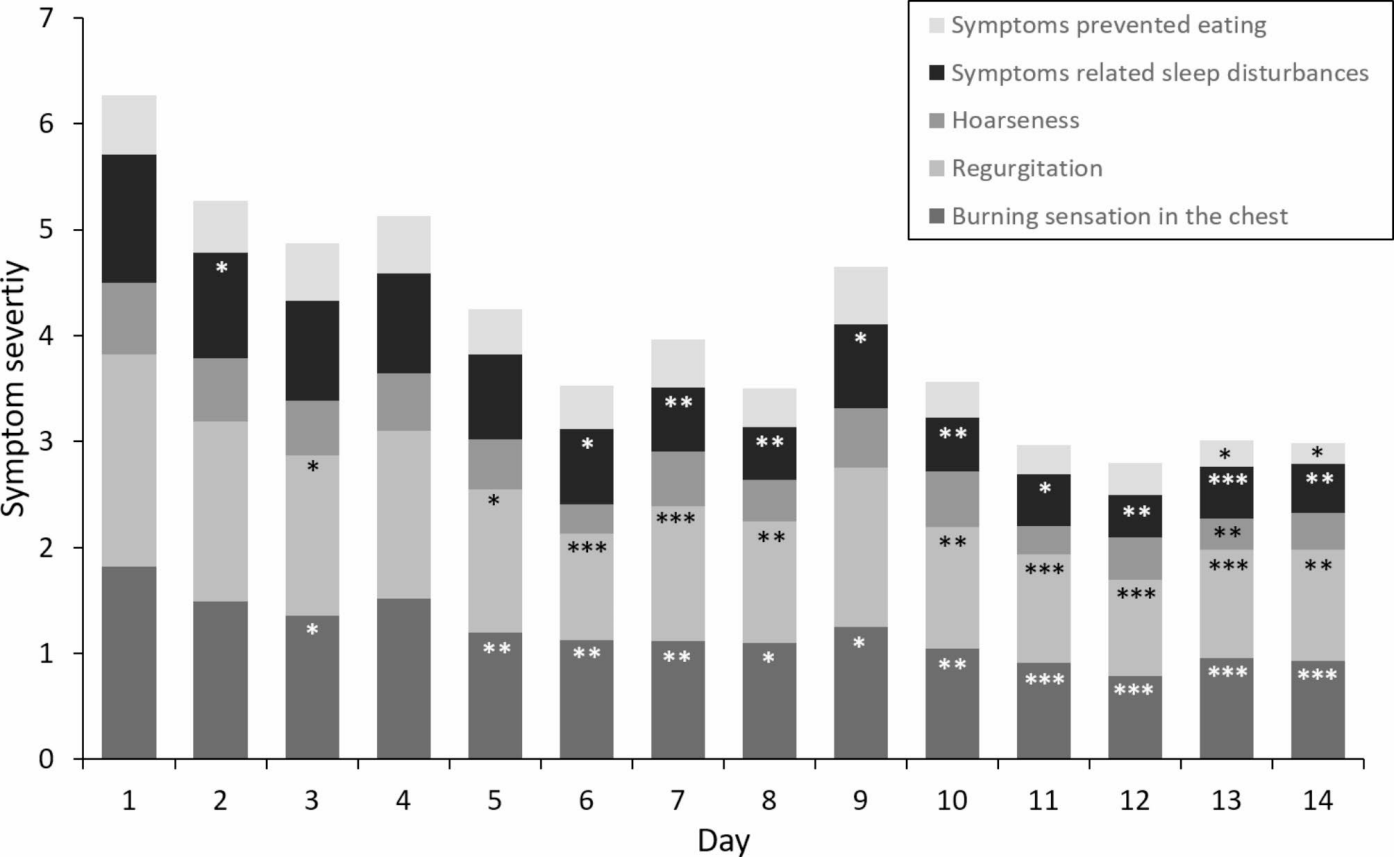
Stack bars of self-assessed daily symptom severity reported by patients in diaries during the two weeks treatment period, severities of individual symptoms assessed on 5-point Likert times are summed up; Significant differences compared to day 1 are marked by stars, *: p < 0.01, **: p < 0.001, ***: p < 0.0001; N = 30–33.

The course of self-assessed symptom severity, based on daily entries in patients’ diaries, is given in Fig. 3. Self-assessed severity of “heartburn” declined from initial 1.8 ± 0.3 to 0.9 ± 0.3 after 14 days whereby improvement was most pronounced within the first days of treatment and differences to day 1 were significant from day 5 onwards (p < 0.01). Similarly, severity of all other assessed symptoms was alleviated by about 50% during the study period with highest improvement of > 60% for parameters “difficulties getting sleep” and “symptoms prevented eating” (Fig. 3).

**Fig. 4:**
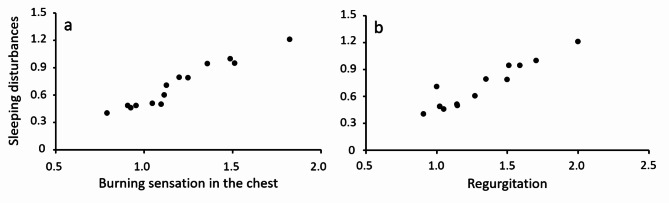
Linear correlations of self-assessed daily mean symptoms severities of “sleeping disturbances“ with “Burning Sensation” (a) and “Regurgitation” (b) reported by patients in diaries during the two weeks treatment period.

Compared to the self-assessed severity at day 1, significant reductions for “regurgitation” (p < 0.05) and “difficulties getting sleep” (p < 0.05) were reported from day 5 and 6 onward, respectively. Symptoms like “hoarseness” (day 13, p < 0.01) and “symptoms prevented eating” (day 13 + 14, p < 0,05) with low initial severity were significantly reduced only in the end of the study period. During the course of the study, linear correlations of daily mean severities of “sleeping disturbances” with “burning sensation” (r^2^: 0.94) and “regurgitation” (r^2^: 0.89) were present (Fig. 4).

Onset of symptom relief was reported to be on average within 15 min for 49% of patients and up to 30 min for another 32% of patients with a calculated mean of 21 min until relief. The mean duration of symptoms relief after intake of the tablet was 3.5 hours until symptoms reappeared. Patients took on average 3 tablets per day corresponding to an overall treatment compliance of 82.2%.

73.8% of investigator ratings on the treatment effectiveness were positive which was confirmed by the ratings by patients (71.4%). Similarly, 73.8% of both, investigators and patients, were satisfied with the add-on therapy. Ratings of effectiveness and satisfaction on Likert items were highly correlated for both investigators (r^2^: 0.68) and patients (r^2^: 0.65).

Data on satisfaction as well as effectiveness were used for calculation of MID. MID estimates based on the mean improvement of symptom severity in patients with minimal positive ratings of treatment effectiveness were 0.91 points, which corresponds to an improvement of 38.6%. This was similar to estimates based on symptom frequency (0.89 points or 36.4%). In comparison, in patients who perceived no effectiveness of treatment, symptoms improved by 0.15 and 0.27 (5.2 and 9.3%), respectively. Similar estimates were obtained based on treatment satisfaction. Thus, the average improvement achieved by patients during the treatment period exceeded MID.

In patients with negative or neutral ratings of treatment, the symptoms sleeping disturbances and sore throat did not improve and were unchanged or even worsened whereas all other symptoms improved during the study period. These two worsened symptoms had pronounced effects on average change of symptoms. In contrast, MID analyses using the RDQ scores yielded no significant differences between patient groups without and with positive ratings of effectiveness (1.1 vs. 1.3 points) corresponding to 32.3 and 36.9%, respectively.

### Safety and tolerability

No SAE or treatment emergent AE were reported during the 2 weeks treatment period. In 98% of all cases, investigators and patients, respectively, rated the tolerability of the product as very good or good.

## Discussion

This non-interventional study investigated the effectiveness of a medical device in patients suffering from persistent reflux after PPI treatment for at least one year. Persistence of GERD can result from several causes, but may severely impact the quality of life and therefore requires adequate treatment [22]. Whereas antacids and PPIs efficiently regulate the acidity of reflux, they fail to control its non-acidic components like pepsin or bile acids from duodeno-gastroesophageal reflux which consequently may still result in (extra-)oesopagheal damages [23, 24]. In particular, pepsin reactivated after endocytosis into laryngeal epithelial is under suspicion to play a central role in extra-oesophageal manifestations and its concentration is potentially even elevated by PPI treatment [25]. Thus, a protective coat not only covering the oesophagus but the laryngeal epithelium as well, as potentially formed by the IMD, can be a valuable contribution to treatment of reflux and prevention of its potential detrimental long-term effects [26]. Furthermore, the xylitol component of the IMD induces salivation whereby the saliva itself is suggested to protect the oesophageal mucosa and alleviates GERD symptoms [27].

In this study, treatment with the medical device resulted in a pronounced symptom relief of severity and frequency for 8 of the 9 symptoms assessed by investigators. In accordance, the symptoms derived subscales of the RDQ and the related GERD Score improved similarly. The reductions of self-assessed symptom severity reported in patient diaries became evident already within the first days of the study. Significant reductions of severity reported by patients for the symptoms “burning sensation in chest” and “regurgitation” were already observed from day 5 onwards. Beneficial effects were observed independently from the PPI applied and extend of improvement agrees with results obtained for chondroitin-sulphate based products which apply a comparable mode of action [28–30]. Chondroitin-sulphate based products have been shown to protect the oesophageal mucosa from damages induced by reflux in ex-vivo experiments [31, 32]. In summary, these studies suggest that a protective coat of the irrigated mucosa supports symptom relief likely by re-epithelisation of affected areas which is supported by the contained hyaluronic acid [33].

With the decline of heartburn and regurgitation severity reported in patient diaries during the 2 weeks study period, sleep disorders were reduced in a secondary fashion. As nocturnal reflux events and resulting sleep disturbances are known to severely affect quality of life, alleviation of symptoms is expected to be highly beneficial for overall wellbeing and the quality of life [34]. Patients who were unsatisfied with treatment or considered its effectiveness as low were characterized by persisting sleep disorders. This suggests, that perception of treatment success may not be equally affected by all GERD symptoms and the average improvement but disproportionally depends on one particular symptom like sleep quality.

This study has its limitations as it was conducted using an open-label and uncontrolled design without adaption for multiple testing. Thus, the improvement of symptoms in this study results cannot be placed in context to similar marketed products or a placebo effect. A potential second limitation was use of different PPIs by patients during the trial and missing records of potentially changed patients’ compliance to PPI. The 2 weeks study duration did not allow assessments of long-term effectiveness and safety/tolerability as well as QoL. Finally, the small sample size only allowed to detect ADEs as well as device deficiencies with high incidences whereas rare events might not be noted. However, the low risk product characteristic and the absence of any adverse events suggest that application is most likely be considered as safe.

Persistent GERD following PPI treatment is a heterogenous condition including among others reflux hypersensitivity, functional heartburn, or insufficient gastric acid suppression by PPI. Future studies with Sobrade® in well characterised patients suffering from GERD despite long-term PPI treatment may help to establish evidence-based therapies for these patients.

## Conclusions

The study revealed that the tested medical device can serve as a valuable add-on in PPI therapy of GERD as treatment resulted in a pronounced improvement of symptoms which was considered by patents as clinically relevant. The rapid onset of symptom relief within minutes and duration of relief of about 3 hours, suggests that Sobrade® may represent a treatment option for mild GERD symptoms on its own and allow partial tapering of long-term PPI use.

### Electronic Supplementary Material

Below is the link to the electronic supplementary material

Supplementary Material 1

## Data Availability

The data that support the findings of this study are available from the corresponding author upon reasonable request.

## Abbreviations

ADE: adverse device effect
BOCF: Baseline observation carried forward
CRO: Contract Research Organization
eDiary: electronic diary
FAS: full analysis set
GERD: gastroesophageal reflux disease
IMD: investigational medical device
MID: minimal important difference
PPI: proton pump inhibitors
RDQ: Reflux Disease Questionnaire
