# Supplementary material for: Multicentre prospective study to evaluate effectiveness and safety of gel-forming and hyaluronic-acid containing chewable tablets as add-on treatment in patients with gastroesophageal reflux disease (GERD) symptoms and unsatisfying proton pump inhibitor therapy
Source: BMC Gastroenterol. 2023 Sep 6;23:304. doi: 10.1186/s12876-023-02946-6 (PMC10483795; doi:10.1186/s12876-023-02946-6)
Supplement: Supplementary file 1 — Supplementary Material 1 [file 12876_2023_2946_MOESM1_ESM.docx]

**Appendix A: Likert Scales used for patient and investigator ratings**

**Table A** Levels of 6-point Likert items used to assess frequencies and severities of GERD symptoms over the past 7 days by investigators during visits

| **Scale level** | **Severity** | **Frequency** |
| --- | --- | --- |
| 0 | Did not have | Did not have |
| 1 | Very mild | Less than 1 day a week |
| 2 | Mild | 1 day a week |
| 3 | Moderate | 2-3 days a week |
| 4 | Moderately severe | 4-6 days a week |
| 5 | Severe | Daily |

**Table B** Levels of 5-point Likert items used for daily self-assessment of symptoms severity by patients in diaries

| **Scale level** | **Self-assess severity** |
| --- | --- |
| 0 | Not at all |
| 1 | Mildly |
| 2 | Moderately |
| 3 | Severely |
| 4 | Not tolerable |

**Table C** Levels of 5-point Likert items used to assess effectiveness of treatment and satisfaction with treatment

| **Scale level** | **Effectiveness** | **Satisfaction** |
| --- | --- | --- |
| -2 | Much worse | Completely dissatisfied |
| -1 | Worse | Somewhat dissatisfied |
| 0 | Unchanged | Neutral |
| 1 | Better | Somewhat satisfied |
| 2 | Much better | Completely satisfied |

**Table D** Levels of 5-point Likert items used to tolerability of treatment

| **Scale level** | **Tolerability** |
| --- | --- |
| -2 | Very poor |
| -1 | Poor |
| 0 | Moderate |
| 1 | Good |
| 2 | Very good |
